# A Review of 10-Year Survivability of Immunotherapy in the Management of Colon Cancer

**DOI:** 10.7759/cureus.43189

**Published:** 2023-08-09

**Authors:** Chiugo Okoye, My Tran, Elizabeth Soladoye, Darlington E Akahara, Chinemerem M Emeasoba, Blessing T Ojinna, Emmanuel Anasonye, Oyindamola O Obadare, Chiamaka S Diala, Bolanle H Salaudeen, Endurance O Evbayekha, Okelue E Okobi

**Affiliations:** 1 Internal Medicine, California Institute of Behavioral Neurosciences & Psychology, Fairfield, USA; 2 Internal Medicine, Baptist Health-University of Arkansas for Medical Sciences - Arkansas, North Little Rock, USA; 3 Internal Medicine, Piedmont Athens Regional, Athens, USA; 4 Medicine, Windsor University School of Medicine, Cayon, KNA; 5 Internal Medicine, Imo State University, College of Medicine, Owerri, NGA; 6 Internal Medicine, Texila American University, Georgetown, GUY; 7 Internal Medicine, All Saints University School of Medicine, Roseau, DMA; 8 Health/Biomedical Informatics, The University of Texas Health Science Center, Houston, USA; 9 Internal Medicine, Lagos State University College of Medicine, Lagos, NGA; 10 Internal Medicine, St. Luke's Hospital, Chesterfield, USA; 11 Family Medicine, Medficient Health Systems, Laurel, USA; 12 Family Medicine, Lakeside Medical Center, Belle Glade, USA

**Keywords:** immunotherapy, progression-free rates, survival rates, colon adenocarcinoma, colon gist, colon lymphoma

## Abstract

Colon cancer is one of the most common cancers in the United States of America. In addition to conventional treatment approaches such as surgery, chemotherapy, and radiation for colorectal cancer, immunotherapy has gained recognition over the past few years. However, its effectiveness in colorectal cancer treatment is controversial. Our study investigates the survival and progression-free rates of immunotherapy for different types of colorectal cancer over the last 10 years. We conducted literature reviews from various clinical trials and research studies to evaluate immunotherapy's role in colorectal cancer treatment. We also investigated how it affects clinical outcomes. We discovered a range of effective immunotherapy approaches targeting various growth factors and signaling pathways. These modalities include monoclonal antibodies aimed at growth factors such as epidermal growth factor (EGF), vascular endothelial growth factor (VEGF), hepatocyte growth factor (HGF), human epidermal growth factor receptor 2 (HER2), and downstream signaling pathways such as mitogen-activated protein kinase (MAPK), kirsten rat sarcoma viral oncogene (KRAS), B-raf proto-oncogene, serine/threonine kinase (BRAF), and phosphatase and tensin homolog (PTEN). Additionally, we identified immune checkpoint inhibitors, such as cytotoxic T-lymphocyte-associated antigen 4 (CTLA-4) inhibitors and programmed cell death ligand 1 (PD-L1) inhibitors, as well as target therapy and adoptive cell therapy as promising immunotherapeutic options.

Nevertheless, the application of immunotherapy remains highly limited due to various factors influencing survival and progression-free rates, including tumor microenvironment, microsatellite instability, immune checkpoint expression, and gut microbiome. Additionally, its effectiveness is restricted to a small subgroup of patients, accompanied by side effects and the development of drug resistance mechanisms. To unlock its full potential, further clinical trials and research on molecular pathways in colorectal cancer are imperative. This will ultimately enhance drug discovery success and lead to more effective clinical management approaches.

## Introduction and background

Colon cancer, also referred to as colorectal cancer (CRC), is a type of cancer that affects either the colon or the rectum, both of which are components of the large intestine. Typically, it originates from small growths called polyps that form in the inner layer of the intestine and have the potential to develop into cancer over time. Among the most prevalent cancers worldwide, colon cancer has several types, with adenocarcinoma being the most common, accounting for more than 95% of cases. Adenocarcinomas begin in glandular cells within the colon or rectum. Other less common types of colon cancer include cancer tumors originating from hormone-producing cells in the intestinal tract and gastrointestinal stromal tumors (GISTs) developing in the walls of the colon or intestine. Although rare, lymphomas can also affect the colon [1].

The precise causes of colon cancer often remain unknown, but various risk factors can increase the likelihood of its development. One prominent risk factor is age, as the risk of colon cancer rises with advancing years, with most cases occurring in individuals over 50. However, colon cancer can affect people of any age. Those with a family history of colon cancer and certain genetic conditions such as Lynch syndrome and familial adenomatous polyposis (FAP) are also at a higher risk of developing the disease. Additionally, a history of pre-cancerous polyps or prior instances of colon or intestinal cancer can elevate the risk. Chronic colon inflammatory diseases, such as ulcers or Crohn's disease, have also been associated with an increased risk [1,2]. Lifestyle choices also play a significant role, with habits such as consuming high amounts of red meat, low fiber intake, lack of physical activity, obesity, smoking, and excessive alcohol consumption contributing to the development of colon cancer [3].

Colon cancer poses a considerable health challenge in the United States. According to estimates from the American Cancer Society, between 2023 and 2033, there will be approximately 147,950 new colon and ovarian cancer cases, and around 53,200 individuals will succumb to these diseases. In the past few decades, the incidence and mortality rate of colon cancer has declined, largely due to increased awareness, early detection through screening, and improvements in treatment options. Screening tests, such as colonoscopy, help identify pre-cancer polyps or early-stage cancers when they are more treatment-resistant [4,5]. Despite these advances, colon cancer is still the leading cause of cancer-related deaths. It emphasizes the importance of regular physical examinations, healthy habits, and seeking medical attention for symptoms or risk factors related to colon cancer. Consultation with a healthcare professional is important to provide personalized information and guidance on colon cancer because they can provide the most current and accurate information according to the individual's situation [5].

## Review

Methodology

The methodology employed for this systematic review adhered to the PRISMA 2020 guidelines. Multiple databases, including PubMed, Cochrane, and Google Scholar, were utilized to ensure a comprehensive literature search. Various keywords and search string combinations were employed, employing the logical operators AND/OR to refine the search results. The search encompassed studies conducted up until May 17, 2023, ensuring the inclusion of the most up-to-date information available.

The specific keywords used for the literature search included "Colon Lymphoma," "Colon GIST," "Colon Adenocarcinoma," "Immunotherapy," "Survival rate," and "Progression-free rate."

These keywords were selected to capture relevant studies about the specified areas of interest, such as different types of colon malignancies, the application of immunotherapy, and important clinical outcomes such as survival and progression-free rates.

By employing a rigorous methodology based on PRISMA 2020 guidelines, conducting a comprehensive literature search, and selecting appropriate keywords, this systematic review aimed to gather and synthesize the most relevant and current evidence available on the topics of interest. Table 1 contains the search strategy used, databases assessed, and the number of articles retrieved.

**Table 1:** Database search strategy and collection

Search strategy	Database	Number of articles
(“Colonic Neoplasms/classification" OR "Colonic Neoplasms/drug therapy” OR "Colonic Neoplasms/immunology" )	PubMed (MeSH)	435
Colon cancer AND Immunotherapy AND Future AND Human	Google scholar	205
Survival rate AND Progression free rate AND colon cancer	Cochrane	383
Total number of articles found		1,023
Number of articles after removing duplicates		773

Eligibility criteria

Inclusion Criteria

Literature written and published in English within the last 10 years using human subjects only and freely available full text were used for this review.

Exclusion Criteria

Gray literature was excluded from this research; the same applied to articles with participants on prior colon cancer treatment and articles not written in English.

Selection process

The selected articles were exported to EndNote, a reference management software, and transferred to an Excel sheet to facilitate further analysis and organization. Each article underwent initial screening based on titles and abstracts to streamline the screening process, allowing for a preliminary relevance assessment. In cases where conflicts arose regarding the eligibility of particular articles, the concerns were addressed through discussions among the co-authors, ultimately reaching a mutual agreement. Following this, the shortlisted articles underwent a comprehensive evaluation by examining the full text to ensure their suitability for inclusion. During the evaluation stage, rigorous inclusion and exclusion criteria were applied to ensure that only articles meeting the predefined criteria were included in the final selection. This meticulous process aimed to maintain the integrity and quality of the review by considering articles that were most relevant and aligned with the research objectives.

Quality assessment

The articles on the shortlist underwent quality assessment utilizing relevant appraisal tools, with the participation of all co-authors in the process. The Newcastle-Ottawa tool was employed to evaluate observational studies to determine their quality. On the other hand, systematic reviews were assessed using the Assessment of Multiple Systematic Review (AMSTAR) tool. The Scale for the Assessment of Narrative Review (SANRA) was utilized to evaluate narrative reviews. This rigorous approach helps ensure that the findings and conclusions drawn from the systematic review are based on sound and trustworthy evidence.

Data collection

**Figure 1:**
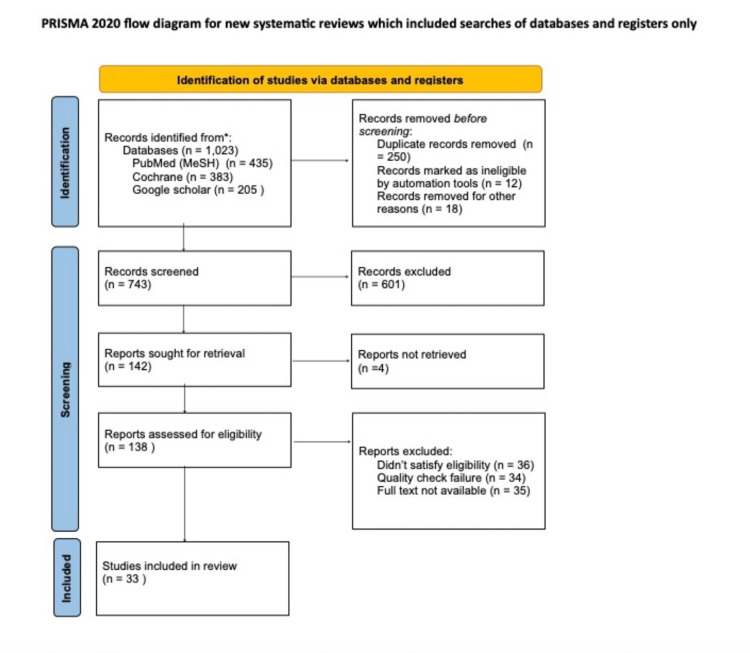
PRISMA flowchart showing the article selection process PRISMA, Preferred Reporting Items for Systematic Reviews and Meta-Analyses

Figure 1 shows the PRISMA flowchart data selection and extraction of articles for this systematic review, the evaluation of primary outcomes, and relevant information. Data collection was conducted independently by eight selected authors, whereas all authors participated equally in reviewing the extracted data and the observed outcomes as per the data extraction questionnaires.

Mechanism of action and role of immunotherapy in the treatment of colon cancer

Various classes of immunotherapy have been identified for the treatment of colon cancer as it is an innovative approach utilizing the body's immune system to recognize and attack cancer cells. The mechanism of action and role of immunotherapy in the treatment of colon cancer can be explained as follows.

Immune Checkpoint Inhibitors

A significant milestone in the field of CRC was the approval of immune checkpoint treatment in 2017 for cancers with deficient mismatch repair (dMMR) or high microsatellite instability (MSI-H) and high mutational burden. However, the current immune checkpoint inhibitors (ICIs) have limited effectiveness against cancer cells with proficient mismatch repair (pMMR), microsatellite-stable (MSS), or low microsatellite instability (MSI-L). This resistance to the immune response is thought to be related to low tumor mutation burden and insufficient infiltration of immune cells. The nobel prize in physiology or medicine in 2018 recognized the discovery of immunological checkpoints, such as programmed cell death protein 1 (PD-1) and CTLA-4, and their significance in cancer immunology [6].

T-cells rely on major histocompatibility complex (MHC) molecules presented by antigen-presenting cells (APCs) in identifying and eliminating cancer cells expressing abnormal antigens due to genetic and epigenetic changes. Checkpoint proteins like CTLA-4, PD-1, glucocorticoid-induced TNFR-related (GITR) protein, and lymphocyte-activation gene 3 (LAG-3) are upregulated in activated CD4+ T-cells, including regulatory T-cells (TREGs) and CD8+ T cells. This dual-check system is vital in effectively combating abnormal cells while preventing excessive immune responses. TREG cells are produced from naïve and effector T-cells through T cell receptor (TCR) stimulation in the presence of transforming growth factor beta (TGF-β) and interleukin 2 (IL-2), resulting in the expression of forkhead box P3 (FOXP3). They play a role in reducing the immune system's response to self and foreign protein antigens to maintain self-tolerance and homeostasis. TREG also expresses CD25, a component of the IL-2 receptor, and its function is governed by FOXP3, a nuclear transcription factor essential for immune homeostasis. TREG's immune-suppressive mechanisms inhibit costimulatory signals by binding to B7 (CD80 and CD86) expressed on antigen-presenting dendritic cells. This regulates the development and function of dendritic cells and naive T-cell activation [6].

TREG cells employ various immune regulation mechanisms, including IL-2 consumption through high-affinity CD25 IL2 receptors chain, secretion of inhibitory cytokines such as TGF-beta, IL-6, and IL-10, metabolic modulation of tryptophan and adenosine, and direct killing of effector T cells [7]. Immune escape occurs when cancer cells evade host immune response and detection by preventing TREG cell migration into the tumor microenvironment. This phenomenon is commonly observed in various malignancies, including CRC. Therapeutic strategies such as ICIs aim to target these regulatory signals expressed by TREG cells [7].

Cytotoxic T-Lymphocyte Associated Antigen 4 (CTLA-4) Ligand Binding

Both regulatory and naive T lymphocytes contain the membrane glycoprotein CTLA-4, which binds to the same B7 ligand on APCs as CD28. However, after TCR stimulation by an antigen, CTLA-4 is produced and exhibits a higher affinity for binding B7 molecules than CD28. This CTLA-4/B7 interaction reduces T cell response, contributing to immunological tolerance, whereas CD28/B7 interaction stimulates cytotoxic immunity [8]. Drugs targeting CTLA-4 have shown favorable outcomes when administered to CRC tumors with MSI and dMMR. Approximately 15% of sporadic CRCs have defective DNA mismatch repair, and patients with MMR-deficient CRCs have demonstrated better stage-adjusted survival than those with MMR-proficient tumors. Changes in MMR status can lead to MSI, resulting in variations in microsatellite length [6].

Programmed Cell Death 1 (PD-1) Pathway

PD-1 interacts with two ligands, PD-L1 and PD-L2, which are present on the cell surfaces of activated lymphocytes, peripheral tissues, organs, and, to a greater extent, tumor cells. These ligands are also expressed on dendritic cells and macrophages. When T cells express PD-1, they become exhausted and lose their ability to perform their effector functions. The interaction between PD-1 and PD-L1/2 suppresses T cell activation and cytokine release, maintaining immunological homeostasis. This situation leads to adaptive immune resistance, where tumor and stromal cells in the tumor microenvironment downregulate invading T cells. Additionally, cancer cells release many TREG cells, which are attracted to tumor cells by chemokine gradients [9].

The first PD-1 blocker to demonstrate effectiveness against MMR-deficient CRC was the humanized IgG4 antibody pembrolizumab. It received U.S. Food and Drug Administration (FDA) approval in 2017 for the treatment of metastatic CRC (mCRC). According to the KEYNOTE-016 research, pembrolizumab successfully treated dMMR CRC but not pMMR CRC, with only anticancer activity observed in MSI-H CRC patients. The KEYNOTE-164 research, which evaluated pembrolizumab in patients with MSI-H mCRC, yielded encouraging findings. While patients with melanoma responded well to pembrolizumab and ipilimumab combination therapy, insufficient data supports its use in CRC [10]. In 2017, FDA approved nivolumab, a different humanized monoclonal immunoglobulin G4 (IgG4) based PD-1 antibody, for treating dMMR or MSI-H mCRC based on the checkmate-142 trial. Nivolumab and the CTLA-4 inhibitor ipilimumab were also combined in additional research.

Further studies showed that combining nivolumab and the CTLA-4 inhibitor ipilimumab outperformed single-agent immune checkpoint blockade. Patients with mCRC who did not respond to chemotherapy were approved for this combination therapy. Ongoing research evaluates new PD-1/PD-L1 inhibitors such as avelumab, durvalumab, and atezolizumab [7]. Additionally, phase I trials are investigating the safety profiles and potential efficacy of new immune checkpoint targets such as T-cell immunoglobulin mucin-3 (TIM-3), T-cell immunoreceptor with immunoglobulin and immune receptor tyrosine-based inhibitory motif (ITIM) domains (TIGIT), and lymphocyte activation gene 3 (LAG-3) in treating CRC.

Epidermal Growth Factor Receptor (EGFR)-Related Pathway

The EFGR transmembrane receptor belongs to the epidermal growth factor receptor family (EGFR/ErbB). It is part of a group of receptors that includes human epidermal growth factor receptor 1 (HER1), HER2/c-neu (ErbB-2), HER3 (ErbB-3), and HER4 (ErbB-4). All these receptors share common features, such as a cytoplasmic tyrosine-kinase-containing domain, a single membrane-spanning region, and an extracellular ligand-binding region. Upon interaction with ligands, the EGFR pathway is activated, leading to the formation of homodimers or heterodimers, which, in turn, cause autophosphorylation of intracellular tyrosine kinase residues. The activation of the EGFR pathway triggers two main intracellular pathways: the mitogen-activated protein kinase (MAPK) pathway and the phosphatidylinositol 3-kinase- (PI3K-) protein kinase B (AKT) pathway. These pathways activate transcription factors, regulating cellular processes in normal cells, including proliferation, migration, differentiation, and apoptosis [11].

Mitogen-Activated Protein Kinase (MAPK) Pathway

In the MAPK pathway, growth factor receptor bound protein 2 (Grb2) bound to the cytoplasmic tyrosine-kinase domain and Sons of Sevenless (SOSs) activate rat sarcoma-rapidly accelerated fibrosarcoma (RAS-RAF). RAS-RAF activation causes mitogen-activated protein kinase phosphorylation (MAPK or MEK). This then activates extracellular signal-related kinase (ERK) that translocates to regulate transcription factors expression. In the phosphatidylinositol 3-kinase-protein kinase B (PI3K-AKT) pathway, PI3K bound to the phosphorylated heterodimer of Erb-B2 receptor tyrosine kinase 2 (ERBB2), and ERBB3 phosphorylates phosphatidylinositol biphosphonate (PIP2) and converts it into PIP3, which promotes the activation of AKT [9]. Activated AKT stimulates various cell targets important for cellular growth.

Mutations involving the EGFR extracellular domain mutations are seen in glioblastomas and are usually associated with gene amplification. Mutations in the tyrosine kinase domain of EGFR may be seen in lung cancer, frequently linked to increased EGFR gene copy numbers. However, unlike lung cancer and other tumors, EGFR gene mutations are uncommon in colorectal malignancies.

Kirsten Rat Sarcoma (KRAS) Pathway

The KRAS proto-oncogene encodes a protein that initiates the MAPK signaling pathway by binding to guanosine 5′-triphosphate (GTP). In several malignancies, including 30%-40% of colorectal tumors, KRAS somatic mutations are common during the early stages of carcinogenesis. These mutations, primarily missense mutations at codon 12/13, lead to constant activation of the KRAS protein by inhibiting its GTPase activity. This results in uncontrolled downstream signaling, which cannot be halted by antibodies targeting the EGFR receptor [12]. In individuals with mCRC, the presence of the KRAS mutation is a significant predictor of resistance to EGFR-targeted therapy (cetuximab and panitumumab). However, it does not predict the response to conventional chemotherapy.

V-raf Murine Sarcoma Viral Oncogene Homolog B (BRAF) Pathway

A downstream serine-threonine protein kinase in the MAPK signaling pathway is encoded by the BRAF gene known as BRAF. Within colorectal malignancies, BRAF mutations are observed in 5%-22% of cases, with a higher prevalence in microsatellite unstable tumors (40%-52%) compared to microsatellite stable tumors (5%). The most common BRAF mutation reported is a valine-to-glutamic acid conversion (V600E). Coexistence of BRAF and KRAS mutations is not possible. Unlike KRAS mutations, BRAF mutations significantly impact prognosis and survival, and their effect may vary depending on the microsatellite status of CRC. Patients with a BRAF mutation in MSS colon cancer tumors tend to have a worse prognosis than those without the mutation. However, the BRAF status does not seem to affect the prognosis of patients with microsatellite-unstable tumors [7]. For patients with metastatic KRAS wild-type tumors with BRAF mutations, shorter progression-free and overall survival (OS) times are observed. The presence of a BRAF mutation also predicts the response to anti-EGFR treatment. In 5%-15% of patients with metastatic colorectal tumors carrying KRAS wild type at codons 12-13 and BRAF mutations, resistance to anti-EGFR therapy occurs. Another article in this series titled "Impact of KRAS mutations on the management of colorectal cancer" provides further details on the predictive role of BRAF mutations [9].

Phosphatase and Tensin Homolog/Protein Kinase B (PTEN/AKT) Pathway

The phosphoinositide 3-kinase-protein kinase B (PI3K-AKT) system can evade regulation through activating mutations in the phosphatidylinositol-4,5-bisphosphate 3-kinase catalytic subunit alpha (PIK3CA) gene (p110 subunit), loss of PTEN gene function, or activation of AKT. KRAS mutations show a significant correlation with PIK3CA exon nine mutations. PIK3CA mutations are associated with a lower cancer-specific survival rate as a prognostic sign, although this association may be relevant only for individuals with KRAS wild-type tumors. Notably, PIK3CA exon 20 mutations are a reliable indicator of poorer outcomes after cetuximab treatment [13].

PTEN, a protein tyrosine phosphatase enzyme, inhibits PI3K activity by dephosphorylating phosphatidylinositol-3,4,5-triphosphate (PIP3), encoded by the PTEN gene. The PI3K-AKT pathway remains constitutively activated when PTEN is lost. PTEN mutations and loss of heterozygosity (LOH) of the PTEN locus have been observed in 13%-18% and 17%-19% of colon tumors, respectively. In patients with KRAS wild-type tumors, the loss of PTEN protein expression is associated with a shorter OS time. Although PTEN mutations/LOH and MSI status are related, recently published results have been contradictory. Additionally, the inactivation of the PTEN protein may not be a reliable indicator of the efficacy of anti-EGFR therapy [10].

AKT is a crucial downstream effector of PI3K. In a large cohort of CRC patients, Ohue and Nishikawa recently investigated the function of activated (phosphorylated) AKT expression and found that early-stage disease and a good prognosis are associated with p-AKT expression. Furthermore, they demonstrated that p-AKT expression and PIK3CA mutation are related, as expected due to their interaction with the EGFR pathway. However, the prognostic effect of p-AKT expression is independent of PIK3CA mutation. Hence, p-AKT expression may be a favorable prognostic indicator in individuals with CRC [7].

The monoclonal antibody (mAb) cetuximab was introduced as CRC treatment in 1995. Following the BOND study, cetuximab received FDA approval in 2004, as it showed increased progression-free survival (PFS) in patients with a low response to single-agent therapy with IRI. When combined with additional chemotherapies like FOLFIRI, cetuximab also increased OS and PFS. However, when combined with FOLFOX (folinic acid [leucovorin], fluorouracil [5-FU], and oxaliplatin), it did not show similar improvements in PFS or OS, likely due to changes in dosage and the impact of CRC molecular heterogeneity. In contrast, when paired with FOLFOX in the PRIME trial, the fully humanized antibody panitumumab reduced the risk of hypersensitivity events and increased PFS and OS. Compared to single-agent panitumumab, maintenance therapy using panitumumab plus 5-FU/LV (5 fluorouracil/Leucovorin) increased PFS and OS, as shown by the VALENTINO study. Cetuximab and panitumumab were equally effective in the phase III ASPECCT study for first-line therapy of CRC. However, anti-EGFR drugs are not a top priority for second-line therapy, as they have shown modest efficacy in multiple studies [14].

Vascular Endothelial Growth Factor (VEGF) Pathway

Angiogenesis, the process of forming new blood vessels or modifying existing ones, plays a crucial role in tumor development, metastasis, and progression. Various proangiogenic and antiangiogenic factors regulate this intricate process. The vascular endothelial growth factor (VEGF) receptors (VEGFRs), consisting of VEGFR-1, VEGFR-2, and VEGFR-3, along with coreceptors NP-1 and NP-2, interact with members of the VEGF family, including VEGF-A, VEGF-B, VEGF-C, VEGF-D, and placental growth factor (PIGF). While VEGF-C and VEGF-D primarily influence lymph angiogenesis, VEGF-A, VEGF-B, and PIGF predominantly contribute to angiogenesis. VEGFR-3 is expressed in lymphatic endothelial cells, whereas VEGFR-1 and VEGFR-2 are mainly found in vascular endothelial cells. VEGFR-1 is believed to regulate angiogenesis by controlling cell migration and differentiation and promoting the differentiation of epithelial cells during early vascular development.

VEGFR-2 is predominantly expressed in blood and lymphatic endothelial cells. When VEGFR-2 binds to VEGF-A, it activates tyrosine residues and several signaling pathways, including phospholipase C (PLC) and RAS/RAF/ERK/MAPK, which stimulate epithelial cell proliferation and inhibit cell death through the PI3K/AKT pathway. Activation of the PI3K and MAPK pathways also triggers changes in adhesion molecules, such as cadherins and catenins, leading to reduced intercellular connection stability, remodeling of the cytoskeleton in epithelial cells, and increased vascular permeability. VEGF-C and VEGF-D activate VEGFR-3, activating the PI3K-AKT/PKB pathway and the RAS/MAPK/ERK pathway. These pathways promote differentiation, migration, proliferation, and survival of lymphatic endothelial cells. While the level of VEGFR-3 expression in tumor cells remains a topic of debate, tumors with lymphatic metastasis often show elevated levels of VEGF-C and VEGF-D, which may contribute to cancer migration through lymphatic capillaries.

In CRC patients, elevated VEGF levels and increased VEGFR activity are frequently observed. Early stages of colorectal neoplasia tend to have higher VEGF levels, especially during metastasis. Neovascularization in metastatic regions and tumor development and migration depend on the proangiogenic actions of VEGF-VEGFR. To target VEGF-mediated pathways in CRC treatment, various VEGF inhibitors are used, including bevacizumab, regorafenib, aflibercept, ramucirumab, and tyrosine kinase inhibitors. These agents function by directly binding to VEGF-A or blocking the extracellular binding region of the relevant receptors. Bevacizumab, for instance, binds to all VEGF-A isoforms, whereas aflibercept acts as a soluble decoy receptor, binding to VEGF and preventing the activation of endogenous receptors. Ramucirumab specifically binds to the extracellular domain of VEGFR-2 with high affinity, inhibiting VEGF ligand binding and receptor activation. Tyrosine kinase inhibitors, on the other hand, block the kinase domains of various receptors involved in angiogenesis once they are internalized in the cell. These treatments aim to disrupt the VEGF-VEGFR signaling axis, thereby hindering tumor angiogenesis and growth [9].

Mesenchymal-Epithelial Transition Factor/Hepatocyte Growth Factor (MET/HPF) Pathway

The mesenchymal-epithelial transition factor (c-MET or MET) and hepatocyte growth factor (HGF) signaling pathways play crucial roles in tumor development, survival, metastasis, and acquired treatment resistance. HGF is the sole known ligand for MET and is mainly released by mesenchymal tissues. MET is expressed in both healthy and cancerous epithelial and endothelial cells, and its overexpression is linked to poor prognosis in CRC. When HGF binds to the membrane-bound MET receptor, it activates the MET signaling pathway. This leads to the activation of downstream signal transduction pathways, including MAPK/ERK, PI3K/AKT, and signal transducer and activator of transcription and janus kinase (STAT/JAK), which control hematopoiesis, organ regeneration, and wound healing. There is an overlap in molecular signaling between downstream pathways activated by EGFR and MET, which may result in compensation for one pathway when the other is inhibited. Other elements, such as the plexin B family of proteins and metastasis-associated in colon cancer 1 (MACC1), can also influence the HGF/MET signaling pathway. In the same cancer tissues, the MET pathway frequently interacts with other receptor tyrosine kinases (RTKs), including EGFR [15].

Clinical trials have shown positive benefits for two mAbs, rilotumumab and ficlatuzumab. Rilotumumab combined with CAP demonstrated prolonged median PFS and OS in gastric or gastroesophageal cancer patients with MET overexpression. However, phase III studies were terminated early due to increased disease-related deaths, emphasizing the importance of patient stratification based on MET expression levels using immunohistochemistry (IHC) or fluorescence in situ hybridization (FISH). A combination of rilotumumab and panitumumab did not show significant advantages in patients with MET-high disease compared to MET-low disease in a randomized trial of patients with KRAS-wild-type mCRC. Phase I trials of ficlatuzumab and TAK-701 are also underway for advanced solid tumors and lung cancer [15].

Competing substances that bind to MET rather than HGF cause atypical dimerization and degradation of MET. Several antibodies, such as onartuzumab, DN-30, and ABT-700, have been developed. Onartuzumab, a mouse-derived mAb with high specificity for the MET semaphorin domain, has been tested in clinical trials for various solid tumors, but results have not been significant in some cancers. DN-30, an antibody that binds to the MET's IPT (immunoglobulin-like plexins transcription factor domain), shows potential to inhibit the spread of MET-positive metastatic melanoma and gastric cancer. ABT-700, a humanized antibody, has completed phase I clinical trials for several solid tumors and has demonstrated tumor regression in preclinical cancer models with MET amplification [14].

Tivantinib is a selective RTK inhibitor that slows the growth of various tumors, but its efficacy in treating CRC has not been determined. AMG 337, an oral ATP-competitive tyrosine kinase inhibitor (TKI) specific to MET, has shown promise in patients with upper gastrointestinal tract cancer with MET amplification. A phase I trial for mCRC investigates the selective MET inhibitor savolitinib, which has shown potential in treating renal cell carcinoma [16]. In patients with EGFR-mutant, MET-amplified non-small cell lung cancer (NSCLC), capmatinib is a useful adjunctive drug to gefitinib. Additionally, non-selective TKIs with FDA approval for certain tumors, such as crizotinib and cabozantinib, have demonstrated antitumor benefits in various cancers.

Clinical trials for CRC explore novel treatments such as tepotinib, foretinib, glesatinib, golvatinib, and sitravatinib. Combination inhibition of MET and EGFR has shown increased PFS in patients with NSCLC with MET overexpression, and targeting HGF/MET may help overcome resistance to EGFR or VEGFR inhibitors [16].

Survival rates and progression-free rate of immunotherapy on colon and rectal cancer

Survival Rates and Progression-Free Rate of Immunotherapy on Adenocarcinoma

There appears to be a paucity of data on immunotherapy use on colon adenocarcinoma. Thus, the following studies relate to the use of immunotherapy in colon cancer. The common medication used are pembrolizumab, nivolumab, and regorafenib. Andre et al. compared pembrolizumab and chemotherapy in microsatellite high advanced colon cancer. The estimated survival rate and progression-free at 12 months and 24 months were 55.3% (95% CI: 47.0-62.9) and 48.3% (95% CI: 39.9-56.2), respectively, in the pembrolizumab group, and 37.3% (95% CI: 29.0-45.5) and 18.6% (95% CI: 12.1-26.3), respectively, in the chemotherapy group [17].

As part of the phase II Checkmate 142 study aimed at assessing disease progression rates in individuals with MSI mCRC, the administration of nivolumab occurred bi-weekly, with the additional inclusion of low-dose ipilimumab every six weeks. Median progression-free and median OS were not reached with a minimum follow-up of 24.2 months (24-month rates were 74% and 79%, respectively). However, clinical response was achieved regardless of baseline demographic and tumor characteristics, including BRAF or KRAS mutation status [18].

**Figure 2:**
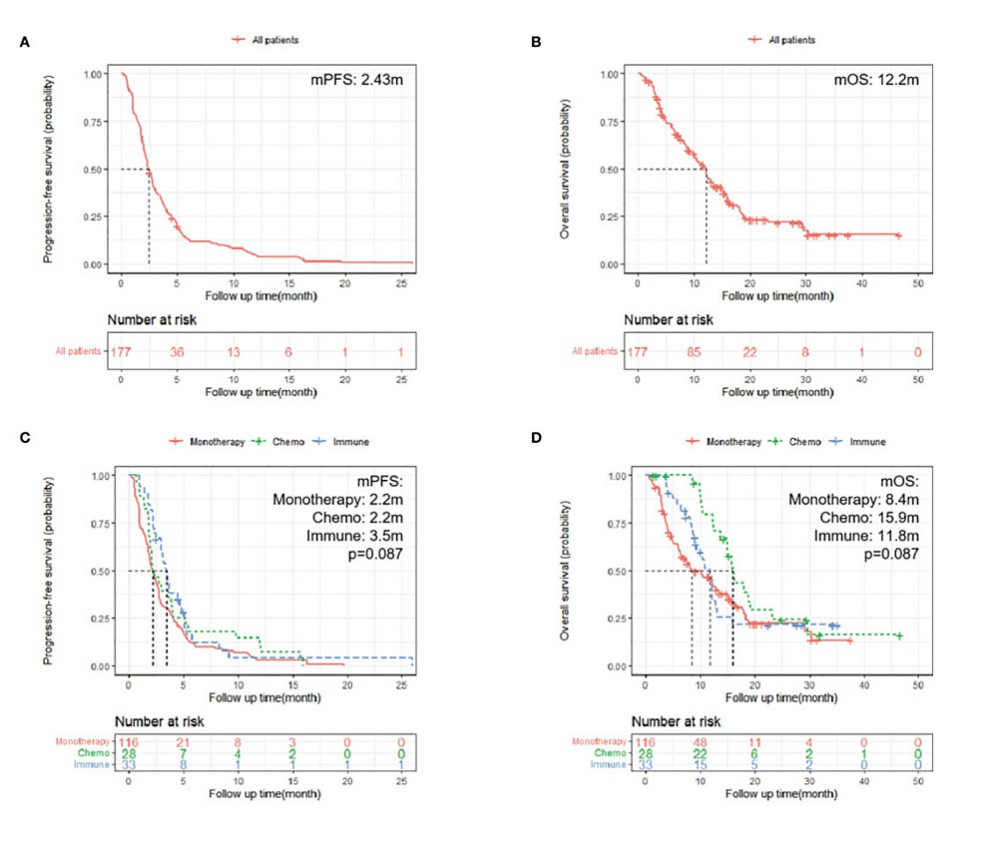
Kaplan-Meier survival curves comparing overall survival and progression-free rate of regorafenib with monotherapy and chemotherapy in the treatment of colon cancer A. A Kaplan-Meier survival curve depicting the progression-free rate of patients used in the study. B. Overall survival analysis of the 177 patients used for the study. C. Progression-free rate of patients categorized into three groups. D. Overall survival of patients categorized into three groups.

A multicenter retrospective cohort study using regorafenib on mCRC compared survival and PFS rates of regorafenib with monotherapy and chemotherapy, as shown in Figure 2 [19]. It revealed that regorafenib administration was associated with a mean PFS of 2.43 months (95% CI: 2.17-2.83) and mean OS of 12.2 months (95% CI: 10.2-13.6) when compared with chemotherapy or monotherapy; PFS in the immune group was significantly better than that in the monotherapy group (3.5 m vs. 2.2 m, HR = 0.65; 95% CI: 0.43-0.99; p = 0.043). However, OS was insignificant (11.8 m vs. 8.4 m; p = 0.37). Although PFS was not significantly longer in the chemotherapy group compared with the monotherapy group (2.2 m vs. 2.2 m; p = 0.25, OS in the chemotherapy group was significantly longer than that in the monotherapy group (15.9 m vs. 8.4 m, HR = 0.57; 95% CI: 0.34-0.95; p = 0.032).

**Figure 3:**
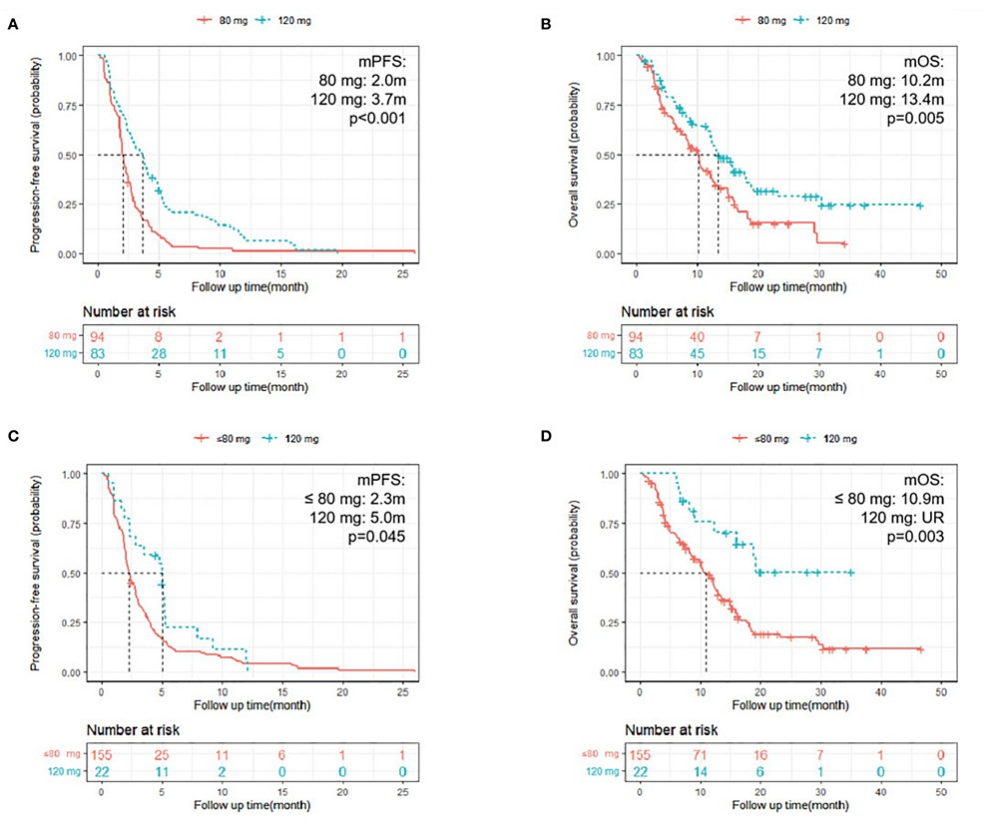
Kaplan-Meier survival curves comparing survival and progression-free rates of colon cancer with 80 mg and 120 mg doses of regorafenib A. The progression-free rate of patients who received different initial doses. B. The overall survival of patients who were administered various starting doses. C. The progression-free rate of patients who received different final doses. D. The overall survival of patients who were given varying final doses.

In terms of the dose of regorafenib administered, patients with a starting dose of 120 mg had longer PFS and OS rate compared with those who began treatment at 80 mg (PFS: mean PFS: 3.7 m vs. 2.0 m; HR = 0.52; 95% CI: 0.38-0.71; p <0.001; OS: mean OS: 13.4 m vs. 10.2 m; HR = 0.59; 95% CI: 0.41-0.86; p = 0.005). Furthermore, patients with a final dose of 120 mg had longer PFS compared with the 80 mg or fewer groups (PFS: HR = 0.61; 95% CI: 0.38-0.99; p = 0.045; mean PFS: 5.0 m vs. 2.3 m; OS: HR = 0.35; 95% CI: 0.18-0.70; p = 0.003; mean OS: UR [unreach] vs. 10.9 m) (Figure 3) [19].

Survival and Progression-Free Rate of Immunotherapy on GIST

The most common type of gastrointestinal mesenchymal soft tissue sarcomas is the GIST. GIST originates from interstitial cajal cells. GISTs are rare tumors, with an estimated incidence of 1.5/100.000 per year, and account for 1%-2% of gastrointestinal neoplasms. The median age of the people affected is around 60-65 years. The most common localization is the stomach (60%) and the small intestine (20%-30%), whereas GISTs are found less frequently in the orthosigmoid and esophagus [20]. Some symptoms of GIST are hemorrhage, anemia, indigestion, and abdominal pain. The National Institutes of Health (NIH) classification system categorizes patients into very low, low, intermediate, and high-risk groups considering the lesion's size and the tumor's mitotic activity [21]. Imaging used for diagnosis includes computed tomography (CT), magnetic resonance imaging (MRI), and positron emission tomography (PET) [21].

Immunotherapy uses medicines to boost the body’s immune response to help fight cancer [22]. The role of immunotherapy in sarcomas is growing fast, with impressive responses being described. The preservation of the proper maturation of the leukocytes makes immunotherapy an attractive adjuvant option in GIST treatment since imatinib, except in rare cases, does not lead to leukopenia [20]. The immune system has two main responses: innate and adaptive. The immune system is also a combination of stimulatory and inhibitory interactions; it is a balancing act that can either drive or inhibit disease progression. Many immunotherapeutic drugs work through the inhibitory mechanisms of the immune system to eradicate tumors or at least slow disease progression [23].

The FDA has approved a few drugs to treat advanced or metastatic GIST. Researchers have introduced precision medicines to target the genetic changes that cause these tumors. For example, mutations in the kit proto-oncogene receptor tyrosine kinase (KIT) or platelet-derived growth factor receptor alpha (PDGFRA) genes are present in the cells of most GISTs. These drugs can affect cells with these gene changes and are often helpful in treating GISTs, but they tend to stop working overtime. These drugs are called targeted therapy, including imatinib (first line), which can be used to shrink a tumor before and after surgery to prevent or delay a recurrence in cases of high-risk GIST. Sunitinib (second line) is used for patients resistant or intolerant to imatinib. The third line of treatment is regorafenib, and the fourth line of treatment is ripretinib [24]. Other drugs targeting the KIT or PDGFRA proteins are also being studied against GISTs. Some of these drugs have been shown to help some patients in early studies, such as sorafenib (nexavar), nilotinib (tasigna), dasatinib (sprycel), pazopanib (votrient), ponatinib (crenolanib), and binimetinib (mektovi).

Imatinib, the first-line immunotherapy for GIST, can achieve a PFS of 1.9 years and a median OS of 3.9 years. It interacts with the immune system at many levels to enhance its antitumor function. In a nutshell, imatinib works by activating CD8+T cells and causing the apoptosis of tumor-infiltrating TREG cells. Although these results are promising, imatinib is not considered curative because secondary KIT mutations tend to develop, causing resistance to the drug [22]. Compared with other mutation types, the prognosis of GISTs with KIT exon 11 mutations is the best. By contrast, GISTs with KIT exon nine mutations must be increased to 800 mg/day because of the low imatinib response [25].

Survival and Progression-Free Rates of Immunotherapy on Lymphoma of the Colon

Lymphoma of the colon, a subtype of non-Hodgkin lymphoma (NHL), is a rare malignancy affecting the lymphatic system in the colon. The highest incidence of colon lymphoma occurs within 50 to 70 years, predominantly affecting males with an approximate ratio of 2:1 [26]. Traditional treatment approaches for lymphoma of the colon have included chemotherapy, radiation therapy, and surgery. However, the emergence of immunotherapy has revolutionized cancer treatment by harnessing the patient's immune system to target cancer cells.

Rituximab's effectiveness in treating gastric and extra-gastric MALT (mucosa-associated lymphoid tissue) NHL has been well-documented, with response rates of approximately 70%. However, it has shown limited durable responses when used as a single agent. To enhance its efficacy, the combination of rituximab with chemotherapy has been explored and has demonstrated synergistic effects, particularly in large-cell lymphomas [27]. Patients with actively progressing gastric MALT lymphoma, following unsuccessful eradication of Helicobacter pylori or experiencing a relapse, have shown remarkable complete response rates (100%) and seven-year event-free survival rates (89.5%) when treated with the combination of rituximab and bendamustine. This treatment approach has demonstrated superiority over the combination of rituximab and chlorambucil [28]. In addition, a novel therapeutic approach known as radio-immunotherapy has emerged, which combines the immunological properties of rituximab, an anti-CD20 mAb, with the targeted radiation effect delivered directly to the tumor site [27].

Other immunotherapies used for colon lymphoma include zevalin, a new compound that includes ibritumomab, a murine parent of the humanized anti-CD20 MoAb rituximab, conjugated by tiuxetan to 90Y [29]. Researchers conducted a study on patients with advanced follicular lymphoma. They had the following results: After a median follow-up of 3.5 years, the findings demonstrated that zevalin treatment significantly extended the median PFS duration for all patients, regardless of whether they achieved a partial or complete response. Patients treated with zevalin had a median PFS of 36.5 months, whereas the control group had a median PFS of 13.3 months. Among patients who achieved a partial response after initial treatment, those who received zevalin had a median PFS of 29.3 months, whereas the control group had a median PFS of 6.2 months. For patients who attained a complete response after initial treatment, the zevalin group had a median PFS of 53.9 months compared to 29.5 months in the control group. Furthermore, 77% of patients who initially had a partial response converted to a complete response, resulting in an overall complete response rate of 87 % [30].

mAbs known as ICIs are designed to counteract the inhibitory effects of T cell molecules, including programmed death receptor-1 (PD-1) and CTLA-4. By blocking these molecules, ICIs enable T cells to identify and eliminate cancer cells more effectively [31], for example, pembrolizumab (Keytruda) and nivolumab (Opdivo) [27]. In clinical studies, it has been observed that using these has led to higher three-year OS rates (58%), as opposed to PD-1 blockade alone (52%) [28]. Antibody-drug conjugates (ADCs) or immunotoxins such as brentuximab vedotin (adcetris) is an antibody targeting CD30, conjugated to a cytotoxic agent used to treat patients with anaplastic large cell lymphoma (ALCL). Moxetumomab pasudotox specifically binds to the CD22 antigen found on specific lymphoma cells and carries a toxin called pseudomonas exotoxin A (PE38) [27].

Factors affecting the response of immunotherapy in colon cancer treatment

Immunotherapy is a cutting-edge approach that harnesses the body's immune system to identify and eliminate cancerous cells. Among the remarkable advancements in this field are ICIs, such as PD-1 and CTLA-4 inhibitors, which have shown remarkable efficacy in various cancers, including colon cancer. Clinical trials like checkmate-142 and KEYNOTE-177 have unequivocally demonstrated the potential of immunotherapy to produce long-lasting responses and improve OS rates in advanced colon cancer cases [32,33].

The introduction of immunotherapy, particularly ICIs, has brought a paradigm shift to oncology, offering a groundbreaking treatment option. However, the effectiveness of immunotherapy in colon cancer is influenced by several factors that significantly impact treatment outcomes. This section delves into these factors, including the tumor microenvironment, MSI, immune checkpoint expression, and the gut microbiome. Understanding these factors is vital for optimizing patient selection and developing strategies to enhance the response to immunotherapy in colon cancer, ultimately leading to improved treatment effectiveness and patient outcomes.

Tumor Microenvironment

The tumor microenvironment plays a critical role in modulating the response to immunotherapy. Factors such as tumor mutational burden, immune cell infiltration, and the presence of immune-suppressive cells influence treatment efficacy. Tumors with high mutational load and increased immune cell infiltration tend to respond better to immunotherapy.

Microsatellite Instability

MSI is characterized by impaired DNA mismatch repair (dMMR) and represents a distinct tumor phenotype. While MSI is found in approximately 5% of mCRC, its prevalence increases to 10%-18% in localized CRC. This phenotype is associated with a high tumor mutational burden, resulting in the generation of immunogenic neoantigens. Consequently, MSI has emerged as a significant predictive biomarker for effective ICIs [34].

Immune Checkpoint Expression

Immune checkpoint molecules, such as PD-1 and its ligand PD-L1, and cytotoxic T-lymphocyte-associated antigen 4 (CTLA-4) regulate immune responses and can be targeted by immunotherapy. Tumors with high expression of these immune checkpoint molecules tend to exhibit better responses to PD-1/PD-L1 and CTLA-4 inhibitors [35].

Gut Microbiome

The gut microbiome has gained increasing attention for its role in modulating systemic immune responses [36]. Recent evidence suggests that specific bacterial species and their metabolites can affect the response to immunotherapy. Certain gut bacteria have been associated with enhanced immunotherapy efficacy, whereas others may hinder treatment outcomes. Modulating the gut microbiome may serve as a potential strategy to improve immunotherapy response in colon cancer.

Other Factors

Additional factors, such as patient characteristics (e.g., age, gender), tumor heterogeneity, and genetic variations, may influence immunotherapy response in colon cancer. Further research is warranted to elucidate their precise impact and potential as predictive biomarkers.

Adverse effects and limitations of monoclonal antibodies and ICIs

mAb therapy targets various signaling pathways, including growth factors such as endothelial growth factor (EGF), VEGF, HGF, HER2, ICIs such as CTLA-4 inhibitors and programmed death-ligand 1 (PD-L1) inhibitors, have gained recognition over the years due to their therapeutic effectiveness in the treatment of CRC [37]. However, they also present adverse effects and limitations.

Hypersensitivity reactions driven by the immune response can occur in CRC patients using mAb therapy. Type I hypersensitivity with anaphylaxis occurred in around 18% of mAb users. Chimeric mAb (cetuximab) and humanized mAb (ex: bevacizumab, onartuzumab, trastuzumab, pertuzumab, camrelizumab, atezolizumab) contain partial murine/rat sequences that can cause immunogenic response. These types of mAbs can cause an immediate anaphylaxis response, even though the actual incident is relatively small. Anaphylaxis reactions mediated by IgE antibodies were reported for cetuximab, trastuzumab, and pertuzumab. Checkpoint inhibitors, including ipilimumab and pembrolizumab, may also cause hypersensitivity pneumonitis, a combination of type III and IV hypersensitivity reactions. Humanized mAb and chimeric mAb can also cause serum sickness-like reactions. mAbs can also cause type IV hypersensitivity reactions such as Steven-Johnson syndrome, dermatitis, rash, and pruritus [38,39].

mAbs are also found to cause non-immune mediate adverse effects. Injection site reactions can include irritation, rash, erythema, swelling, pruritus, pain, and hematoma at the injection sites. Infusion reactions mediated by massive cytokine release can occur with cetuximab, panitumumab, and trastuzumab, especially in the first drug infusion. Anti-cancer mAbs are also found to cause cytopenia. Bevacizumab was reported to cause neutropenia. Severe thrombocytopenia was reported with the use of trastuzumab. Interstitial pneumonitis was reported with cetuximab. Acute respiratory distress syndrome, interstitial pneumonitis, and pleural effusion can occur with trastuzumab. Bevacizumab can cause bronchospasm and pulmonary hemorrhage [38,39]. Cardiac adverse events include cardiopulmonary arrest, heart failure, and cardiomyopathy. Liver adverse events include hepatotoxicity and hepatitis. In addition, checkpoint inhibitors (ex: nivolumab, ipilimumab, pembrolizumab) can cause inflammation of various organs in the body with different frequency and severity. Most common adverse events related to each organ system include maculopapular rash (30%-50%), colitis (20%-40%), hepatitis (4%-15%), hypothyroidism (10%-50%), pneumonitis (1%-5%), arthralgia (<1%-15%), myocarditis (<1%), anemia (<1%-5%), nephropathy (<2%-30%), posterior reversible encephalopathy (<4%-15%), and uveitis (about 1%). Some other adverse events are Sjogren syndrome, conjunctivitis, retinitis, hypophysitis, encephalitis, myasthenia gravis, necrotizing myositis, and Guillain-Barre syndrome [40,41].

Antibody-drug resistance is one of the significant limitations in the treatment of CRC. mAbs targeted against growth factors such as VEGF and EGF have shown limited survival benefits. In patients with mCRC, anti-VEGF therapy was shown to have benefits only for a few months. Patients exposed to bevacizumab (an anti-VEGF therapy) were shown to have decreased VEGF levels and increased levels of VEGF type A receptor 1, leading to drug resistance. Acquired resistance was also observed in patients using anti-HGF therapy (ex: rilotumumab) due to decreased levels of HGF from drug exposure [42]. Other targets of monoclonal therapy are EGFR and downstream signaling pathways, including Kirsten rat sarcoma viral oncogene homolog (KRAS), phosphatidylinositol 3-kinases (PI3Ks), phosphatase and tensin homolog deleted on chromosome 10 (PTEN), and v-raf murine sarcoma viral oncogene homolog B1 (BRAF). Cetuximab (an example of anti-EGFR antibody therapy) has been shown to have an improved OS rate in patients with wild-type EGFR and wild-type KRAS, which only accounts for 10%-20% of all CRC patients. However, 80%-90% of CRC patients who got mutations in EGFR or downstream signal pathway molecules, including KRAS, BRAF, PI3K catalytic subunit alpha (PI3KCA), and PTEN, did not respond well to cetuximab [37]. HER2 is expressed in 3%-5% of patients with mCRC. mAbs that target HER-2, such as trastuzumab and pertuzumab, can also encounter drug resistance due to intrinsic mutation, such as inactivated, truncated HER2 receptors lacking a binding domain for trastuzumab. In addition, any alteration of the downstream signal pathway that involves PI3K mutation or loss of PTEN can also lead to drug resistance to HER2 receptor therapeutic drugs [43].

ICIs that target PD-1 (ex: pembrolizumab, nivolumab, camrelizumab), PD-L1 (ex: atezolizumab, avelumab, durvalumab), and CTLA-4 molecules (ex: ipilimumab, tremelimumab) have shown to have clinical benefit including long-term remissions and OS of CRC patients. However, ICI is only effective in a small subgroup of mCRC patients (about 4%) who were found to have dMMR or MSI-H. Around 96% of mCRC patients have microsatellite stable/DNA mismatch repair proficiency (MSS/pMMR). There is also a lack of biomarkers to predict response outcomes from ICI therapy [44,45].

Other immunotherapy modules and their limitations

Other immunotherapy modules, including adoptive cell therapy (ACT) and cancer vaccine for CRC, have shown limited therapeutic responses. ACT that selects host cells, T cells that are combined with engineered chimeric antigen receptor (CAR), or TCRs that developed anti-tumor properties have been shown preliminary results in clinical trials. However, CAR T-cell therapy has shown limited clinical response due to restricted molecular trafficking, lack of antigen, or low tumor infiltration [46]. Cancer vaccines for CRC have been developed for patients with small lesion residues or as adjuvant therapy at advanced cancer stages. Various study trials on CRC vaccines have been conducted, but none have passed phase III clinical trials. One possible explanation is that the immunity provided by the vaccine did not last long enough to have any clinical impact on patients’ survival rates [47].

Future directions of immunotherapy in treating colon cancer and its clinical importance

Immunotherapy has revolutionized the treatment of colon cancer, offering new hope to patients facing this challenging disease. Here are some future directions of immunotherapy in colon cancer treatment: Combining immunotherapies with other modalities, such as chemotherapy, targeted agents, or other immunotherapies, has emerged as a promising direction in colon cancer treatment. These combinations could improve outcomes and overcome resistance observed in single-agent immunotherapies [48]. Biomarkers identification is crucial for identifying patients likely to respond to immunotherapy. Developing predictive and prognostic markers will serve as a tool for patient stratification. For example, biomarkers such as tumor-infiltrating lymphocyte levels (TILs) and MSI status are already known to predict the response of colon cancer patients to immunotherapy [49]. Recent studies have identified new immune checkpoint pathways such as LAG-3 and TIGIT as important therapeutic targets. As with PD-1 and CTLA-4, mAbs targeting these emerging immune checkpoints are under clinical investigation for colon cancer treatment [50]. The utilization of neo-adjuvant and adjuvant immunotherapy has shown the potential to enhance the treatment of colon cancer. Neo-adjuvant immunotherapies administered before surgery target reducing tumor size and enhance the immune system's ability to clear cancer cells.

On the other hand, adjuvant immunotherapies are expected to improve outcomes for patients who have undergone surgical interventions [51]. CAR-T cell therapy is considered a highly promising strategy among the various cell therapies investigated for CRC. Ongoing preclinical and clinical studies extensively assess CAR-T cells' effectiveness and safety, targeting a diverse range of overexpressed molecular targets in CRC. However, it is essential to note that the clinical development of CAR-T cell therapy for CRC is still in its early stages, primarily consisting of phase I and I/II clinical trials. In addition, it uses genetically modified T-cells to target cancer cells expressing a specific antigen type [52]. These are a few of many research directions in immunotherapy for colon cancer and their clinical importance. For example, immunotherapy-based treatments have changed the treatment of colon cancer with remarkable clinical success, and patients’ survival is set to improve soon. Advancing our understanding of the factors affecting immunotherapy response in colon cancer will facilitate the development of personalized treatment approaches. Integrating molecular profiling, biomarkers, and advanced technologies, such as single-cell sequencing, will aid in identifying patients likely to respond to immunotherapy. Combination therapies targeting multiple immune checkpoints, modifying the tumor microenvironment, and optimizing the gut microbiome hold promise for enhancing immunotherapy efficacy. Immunotherapy represents a promising therapeutic strategy for colon cancer, but its effectiveness varies among patients. In addition, comprehensive assessment and integration of these factors will be essential for personalized treatment selection and optimizing the efficacy of immunotherapy in colon cancer patients.

Limitations

Our study focuses on survival and progression-free rates over the last 10 years of immunotherapy on various CRCs. This retrospective study can underestimate or overestimate the survival and progress-free rates as many drugs are still under phase II and III clinical trials or have recently been approved by the FDA. While current immunotherapy has shown clinical effects and its use either as monotherapy or adjuvant therapy with surgery, chemotherapy, and radiation therapy, its application is still limited due to its potential adverse effects, intrinsic or acquired drug resistance, and therapeutic effects on a small subgroup of patients. Further study of molecular mechanisms of the immune system and cancer cells, including signal pathways, molecular markers, and more drug study trials, are required for better knowledge and application of immunotherapy.

## Conclusions

Colon cancer is one of the leading causes of cancer-related deaths worldwide. Recent advances have resulted in the use of immunotherapy to treat colon cancer. This study found that immunotherapies such as cytokine-based therapies, ICIs such as mAbs, anti-KIT antibodies, and cellular therapies with mAbs are currently being used to treat GIST, adenocarcinoma subtype, and lymphoma. Furthermore, immunotherapy has been shown to improve the survival rate of patients with CRC when compared to conventional chemotherapy. However, factors such as the tumor microenvironment, MSI, immune checkpoint expression, and the gut microbiome impact the response to immunotherapy and its effectiveness. In addition, adverse reactions, including immune-mediated responses such as hypersensitivity reactions to non-immune mediated reactions, antibody-drug resistance, and inadequate therapeutic response, further constrain the effective use of immunotherapy for the treatment of colon cancer. In addition, more research is required to address these issues affecting immunotherapy efficacy to maximize patient benefits, given the increased use of immunotherapy and relatively improved therapeutic advantage over conventional chemotherapy.
